# Unveiling a Small Bowel Obstruction: A Case of a Neuroendocrine Ileal Tumor

**DOI:** 10.7759/cureus.66646

**Published:** 2024-08-11

**Authors:** Clara Leal, Maria Gualter Baptista, Rita Marques, João Pinto-de-Sousa

**Affiliations:** 1 General Surgery, Unidade Local de Saúde de Trás-os-Montes e Alto Douro, Vila Real, PRT; 2 General Surgery, Clinical Academic Centre Trás-os-Montes e Alto Douro, Vila Real, PRT

**Keywords:** gastrointestinal surgery, small bowel obstruction, small bowel neoplasms, gastrointestinal tumors, neuroendocrine tumors

## Abstract

Neuroendocrine tumors (NETs) are rare, slow-growing tumors originating from the diffuse neuroendocrine cell system, predominantly affecting the digestive tract. Small bowel neuroendocrine tumors (SBNETs) may present with nonspecific symptoms, such as abdominal pain, or with intermittent intestinal obstruction. This case outlines the diagnostic journey of a septuagenarian male with prolonged abdominal symptoms and weight loss. Despite extensive investigation, a definitive cause remained elusive. Recurrent partial intestinal obstruction led to surgical exploration and segmental resection. Pathology confirmed a NET. The case underscores the importance of considering intestinal neoplasia in older patients with recurrent partial small bowel obstruction.

## Introduction

The term “neuroendocrine neoplasm” encompasses both well-differentiated neuroendocrine tumors (NETs) and poorly differentiated neuroendocrine carcinomas (NECs) [1,2]. NETs are epithelial tumors arising from neural crest-derived cells, reflecting their predominantly neuroendocrine differentiation [1,3]. The presentation and natural history of NETs vary depending on their site of origin (foregut, midgut, or hindgut) and the hormones they secrete [1,4].

Small bowel neuroendocrine tumors (SBNETs), originating from the midgut, can secrete functional hormones or amines [4]. Although the incidence of SBNETs is low (12 cases per 100,000), it has been increasing over recent decades and has now surpassed adenocarcinoma as the most common primary tumor of the small bowel [4]. This increase may be attributed to diagnostic refinement resulting from advancements in imaging technologies, which are now more widely available [3].

In general, SBNETs exhibit indolent behavior, with patients typically being diagnosed in their sixth or seventh decades of life [3,5]. SBNETs may be asymptomatic at presentation, with incidental diagnoses, or symptomatic, presenting with abdominal pain, intestinal obstruction, gastrointestinal bleeding, and carcinoid syndrome [4,6]. Most SBNETs occur approximately 100 cm proximal to the ileocecal valve [4,7].

Despite the lack of high-quality studies, surgical resection has been identified as the first-line treatment for SBNETs. It can improve patient survival and potentially reduce the risk of developing metastasis and carcinomatosis [4]. Herein, we report the case of a male patient in his 70s who presented to the Emergency Department (ED) with a small bowel obstruction.

## Case presentation

An independent 76-year-old male patient presented to the ED with symptoms of profuse vomiting and an absence of bowel movements, although he was still passing gas. These symptoms had developed and worsened over the past few hours.

His medical history included arterial hypertension, diverticular disease, chronic obstructive pulmonary disease, dyslipidemia, and depressive syndrome. There was no previous history of abdominal surgery. Additionally, the patient had been evaluated at the outpatient clinic for complaints of abdominal distension, flatulence, colicky diffuse abdominal pain, irregular bowel movements, and weight loss, with intermittent and self-limiting episodes of partial intestinal obstruction over the past few months. There was no reported fever, blood or mucus in the stool, flushing, or diarrhea. Exhaustive diagnostic modalities were performed. Initially, abdominal and pelvic computed tomography (CT) and magnetic resonance imaging (MRI) suggested mesenteric panniculitis. Upper and lower endoscopy (with terminal ileoscopy) showed no lesions. Subsequently, to exclude inflammatory bowel disease, a CT enterography was conducted, yielding no significant findings.

In the ED, the patient presented with hemodynamic stability. The abdominal examination revealed distension, tympanic to percussion, without pain or peritoneal signs. Blood tests showed no anemia (Hb 13.6 g/dL), a white cell count of 15.73 x 10³/µL, and no evidence of kidney injury or electrolyte disturbance.

**Figure 1:**
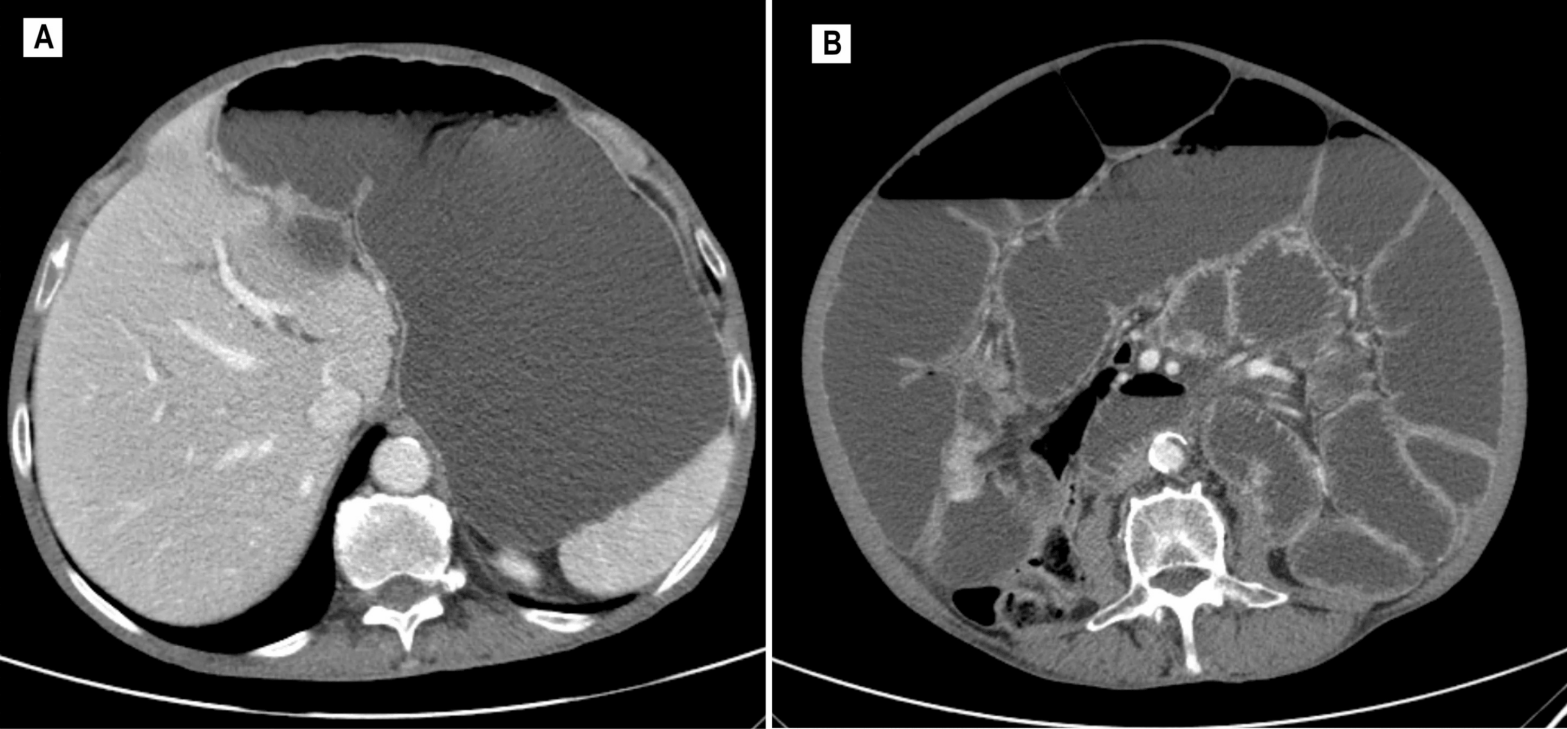
Abdominal and pelvic CT scan displaying notable gastric distention (A) and small-bowel distension (maximum caliber: 74 mm), leading to large bowel collapse (B). CT: Computed tomography

**Figure 2:**
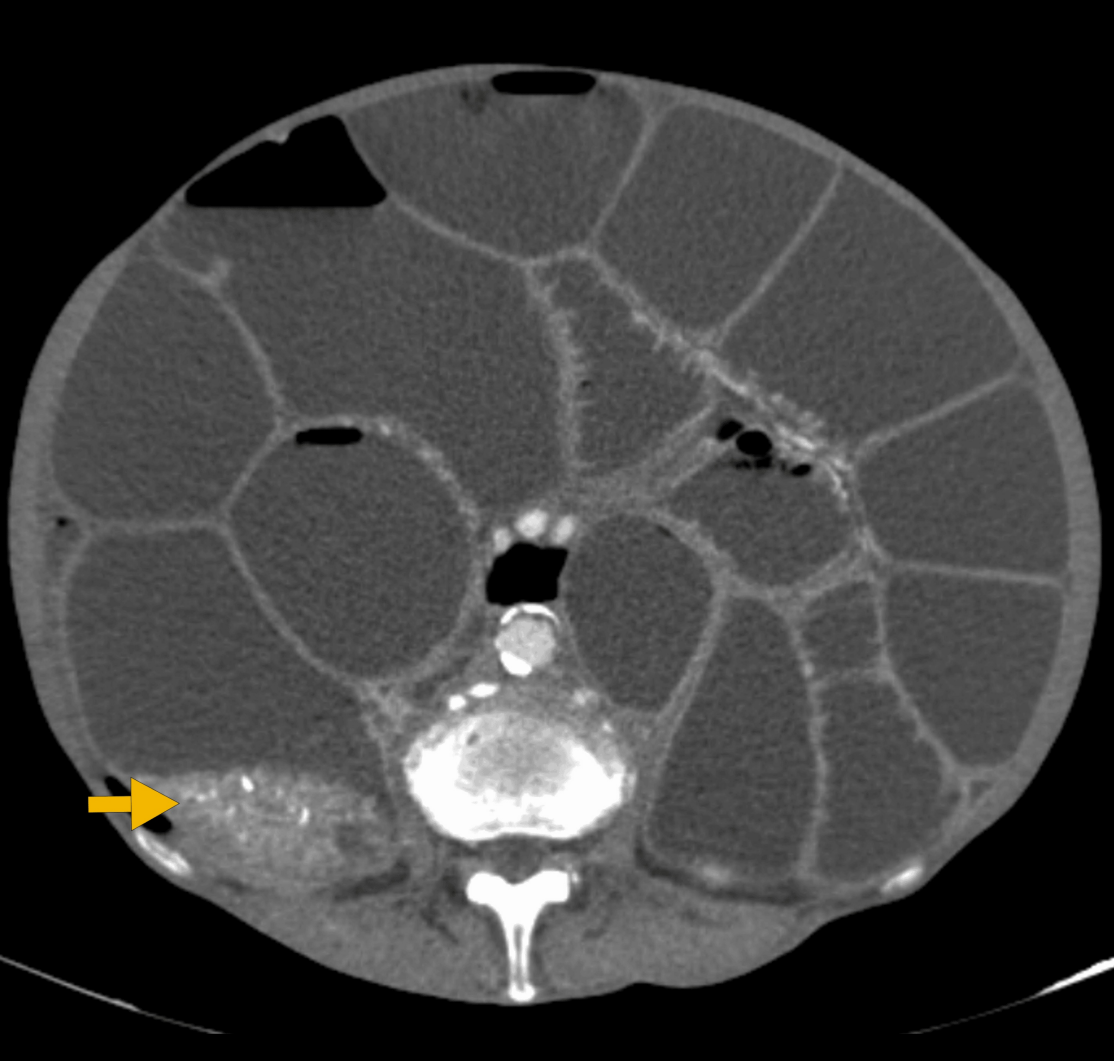
Abdominal and pelvic CT scan revealing hypervascular thickening at the level of the ileocecal valve (arrow), corresponding to the transition point. CT: Computed tomography

Given the patient’s history of frequent episodes of partial intestinal obstruction over the last few months, a second CT scan was performed and revealed a lesion located at the ileocecal valve, suspicious for a NET, angiodysplasia, or gastrointestinal stromal tumor (GIST) (Figures 1-2).

A conservative approach was initiated, consisting of bowel rest, analgesia, prokinetics, fluids, and placement of a nasogastric tube, which revealed abundant enteric drainage. Staging thoracic, abdominal, and pelvic CT scans showed no evidence of distant disease. Tumor markers were negative.

**Figure 3:**
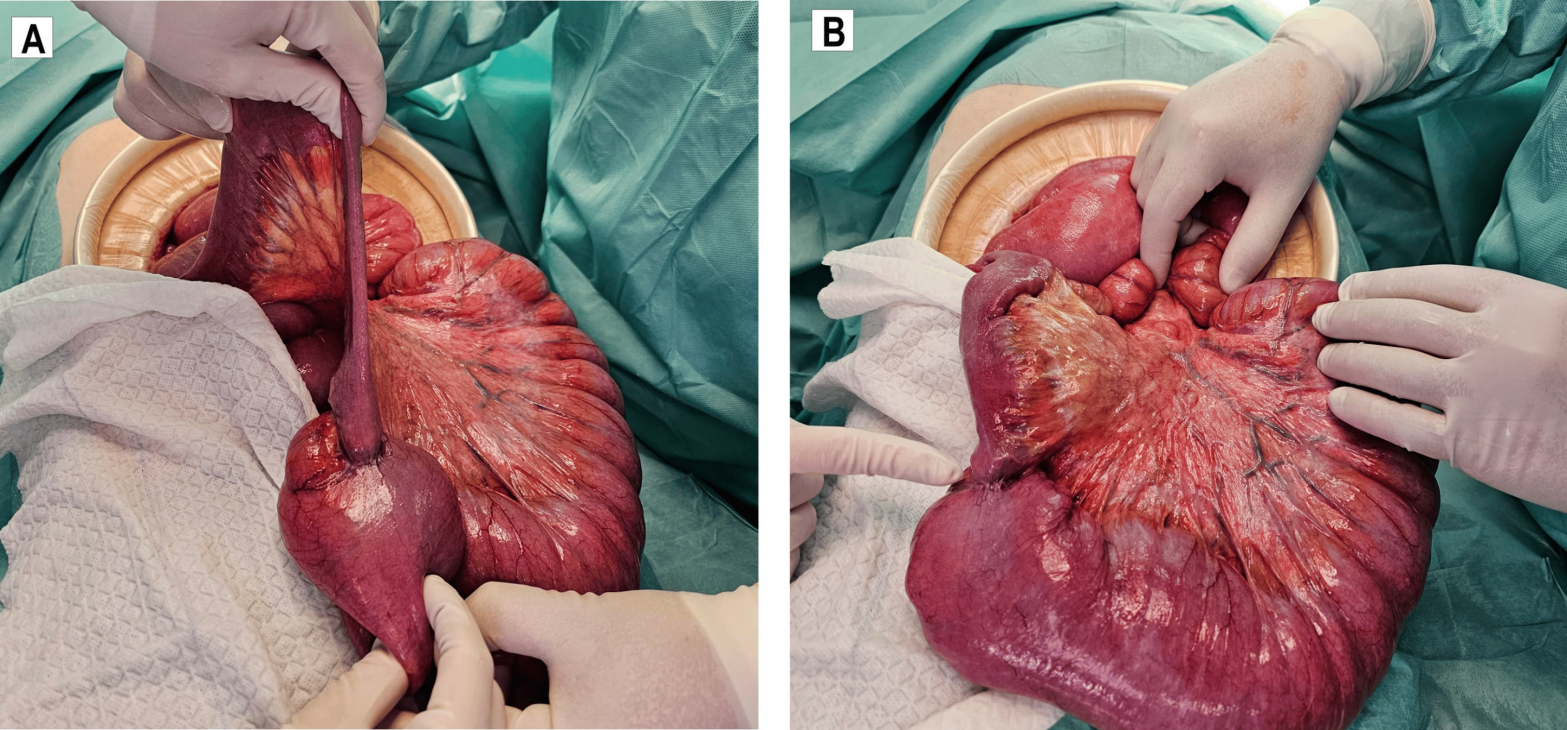
Intraoperative findings A stenotic ileal lesion is located approximately 60 cm proximal to the ileocecal valve, resulting in proximal intestinal distension.

The patient underwent an exploratory laparotomy, which revealed significant small intestine dilation with a transition point at an ileal lesion approximately 60 cm proximal to the ileocecal valve (Figures 3A-3B). Subsequently, a segmental enterectomy was performed, followed by a primary side-to-side anastomosis.

**Figure 4:**
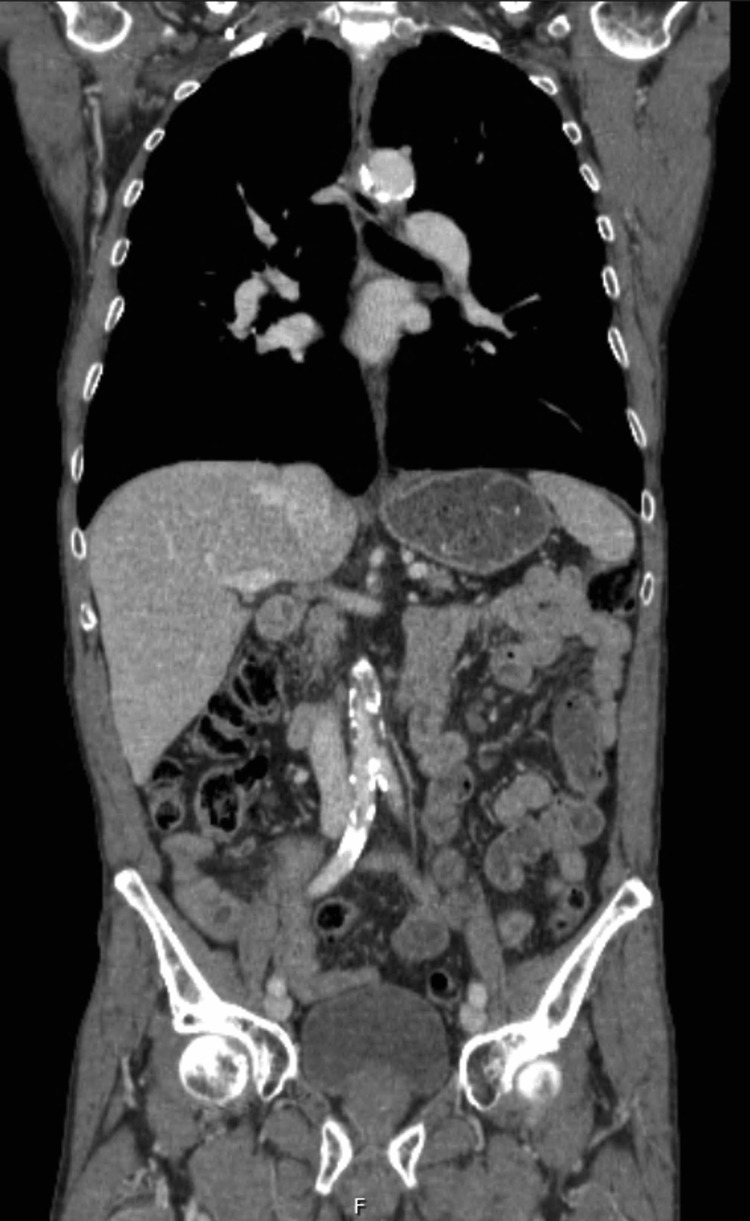
Abdominal and pelvic CT scan at postoperative follow-up shows resolution of the intestinal obstruction, with a normal caliber of the small intestine. CT: Computed tomography

No major complications occurred in the postoperative (PO) period, with discharge on PO day 8. Pathology results indicated a well-differentiated (G1) ileum NET with a greatest diameter of 2 cm. Complete resection was achieved, with the smallest surgical margin measuring 4 cm. A decision for surveillance was made by a multidisciplinary group consult. At the sixth month of follow-up, the patient was asymptomatic, and a repeat CT scan showed no significant alterations (Figure 4).

## Discussion

NETs are slow-growing tumors that arise from the diffuse neuroendocrine cell system distributed throughout the body. They most frequently occur in the digestive system, followed by the lungs [2,8]. Although primary tumors of the small intestine are rare, accounting for only 1-3% of all gastrointestinal cancers, there has been an increasing incidence of SBNETs [8]. Hence, these tumors have recently surpassed small intestine adenocarcinomas in prevalence, and, among the NETs, SBNETs have become the most common gastrointestinal NETs [8].

SBNETs can secrete functional hormones or amines, leading to a clinical condition known as carcinoid syndrome. This syndrome includes symptoms such as diarrhea, flushing, bronchospasm, coughing, or wheezing [3,9]. However, most SBNETs are nonfunctioning [3], and the syndrome, affecting approximately 5-7% of patients, is typically associated with liver metastasis [3,9]. Patients are typically diagnosed in their sixth or seventh decade of life, with males being slightly more affected than females [2,3].

NETs often manifest without noticeable symptoms, but patients may present with abdominal pain, abdominal mass, bowel obstruction, diarrhea, weight loss, and bleeding [3,8]. Abdominal pain is the most common initial symptom, occurring in about 40% of cases, while intermittent obstruction occurs in 25% of SBNETs [10].

Histologic diagnosis is mandatory in all patients, and disease stage and tumor grade are the two major independent prognostic parameters that should always be evaluated [2]. CT is the basic radiological method for imaging NETs due to its wide availability, standardized and reproducible technique, and generally high diagnostic yield [2]. Surgery is the preferred treatment for localized or locoregional disease in NETs with grades G1 and G2 [2].

The case presented involves a male patient in his 70s who experienced a prolonged period of nonspecific symptoms, including abdominal pain, distention, flatulence, weight loss, and irregular bowel movements. Previous endoscopic and imaging studies ruled out malignancy and inflammatory bowel disease. However, worsening symptoms, accompanied by frequent episodes of partial intestinal obstruction, prompted a repeat CT scan, which raised suspicion of a small intestine neoplasm. This clinical presentation aligns with the epidemiology and clinical behavior of SBNETs described earlier. Due to the worsening symptoms and new imaging findings, along with significant intestinal dilation, the patient underwent an exploratory laparotomy followed by resection of the affected segment to relieve the obstruction. Pathology results confirmed a well-differentiated (G1) ileum NET. The patient is currently under surveillance. The five-year survival rate correlates with the disease stage at diagnosis, reaching 65% for patients with localized disease [3].

## Conclusions

SBNETs are uncommon yet increasingly prevalent. This case provides valuable insights into the diagnosis and management of such rare conditions. It underscores the importance of considering and investigating intestinal neoplasms in the differential diagnosis of partial small bowel obstruction, particularly in older patients with no prior abdominal surgeries.
